# Supplementary material for: Landscape and mutational dynamics of G-quadruplexes in the complete human genome and in haplotypes of diverse ancestry
Source: bioRxiv. 2025 Jun 25:2025.06.17.660256. Preprint. [Version 2] doi: 10.1101/2025.06.17.660256 (PMC12262261; doi:10.1101/2025.06.17.660256)
Supplement: 1 [file NIHPP2025.06.17.660256V2-supplement-1.pdf]

bioRxiv preprint doi: https://doi.org/10.1101/2025.06.17.660256; this version posted June 25, 2025. The copyright holder for this preprint (which was not certified by peer review) is the author/funder, who has granted bioRxiv a license to display the preprint in perpetuity. It is made available under aCC-BY 4.0 International license.

Supplementary Material

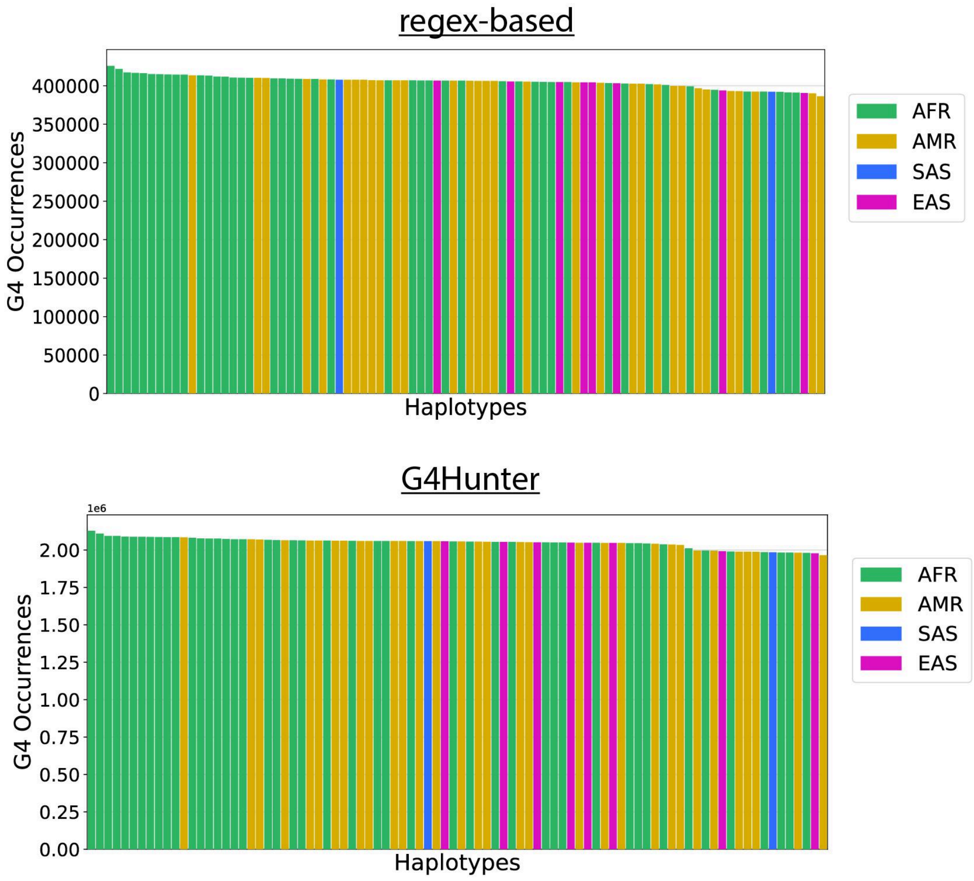

Supplementary Figure 1: Total G4 occurrences per haplotype. Top panel represents G4s from the regex-based algorithm, and the bottom panel from the G4Hunter algorithm. Colors represent the ancestry of each haplotype.

bioRxiv preprint doi: https://doi.org/10.1101/2025.06.17.660256; this version posted June 25, 2025. The copyright holder for this preprint (which was not certified by peer review) is the author/funder, who has granted bioRxiv a license to display the preprint in perpetuity. It is made available under aCC-BY 4.0 International license.

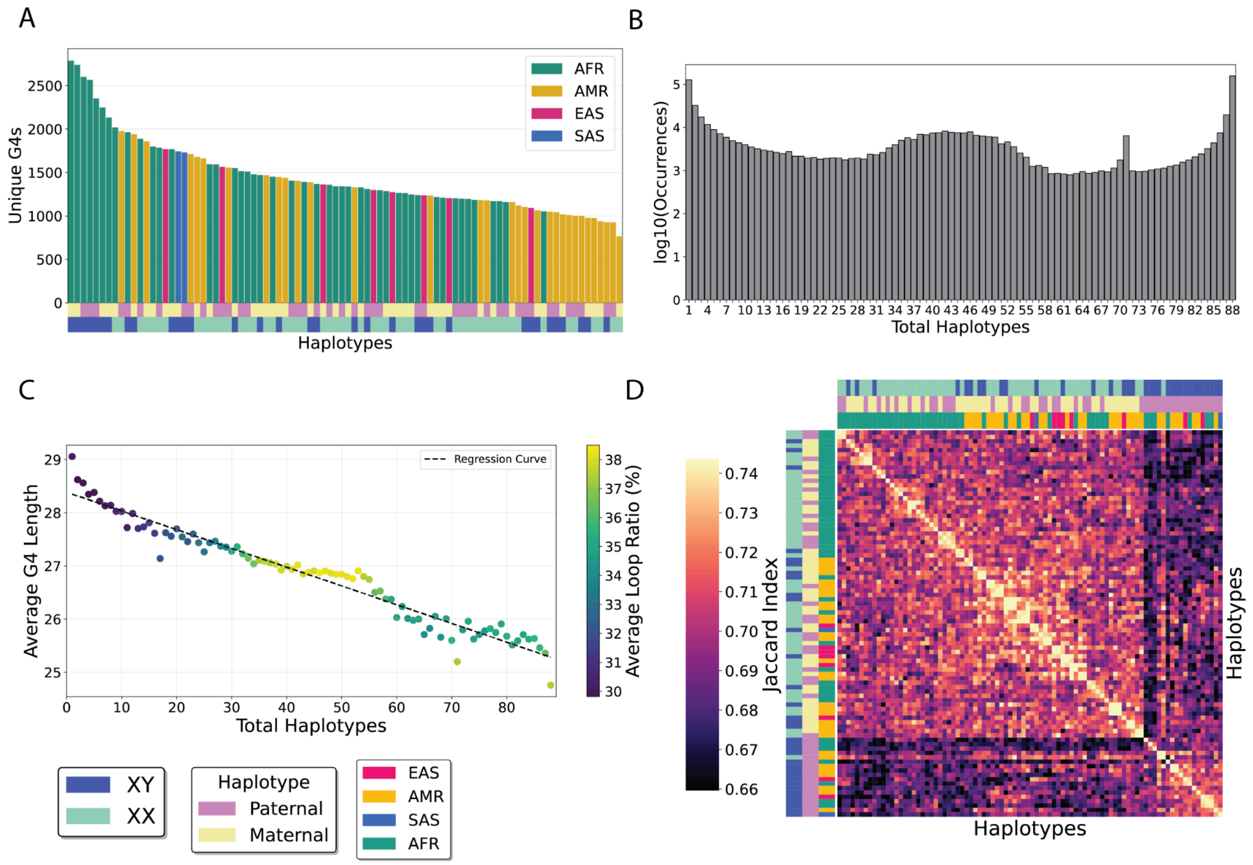

Supplementary Figure 2: **Characterization of G4s across the T2T reference human genome using the regex-based algorithm. A**. Number of unique G4s per human haplotype. **B.** Number of G4s motifs uniquely shared across different numbers of haplotypes. **C.** Number of G4 motifs uniquely shared across different numbers of haplotypes plotted against average G4 length. **D.** Hierarchical clustering of conserved G4s found in the reference genome CHM13v2 across 88 haplotypes.

bioRxiv preprint doi: https://doi.org/10.1101/2025.06.17.660256; this version posted June 25, 2025. The copyright holder for this preprint (which was not certified by peer review) is the author/funder, who has granted bioRxiv a license to display the preprint in perpetuity. It is made available under aCC-BY 4.0 International license.

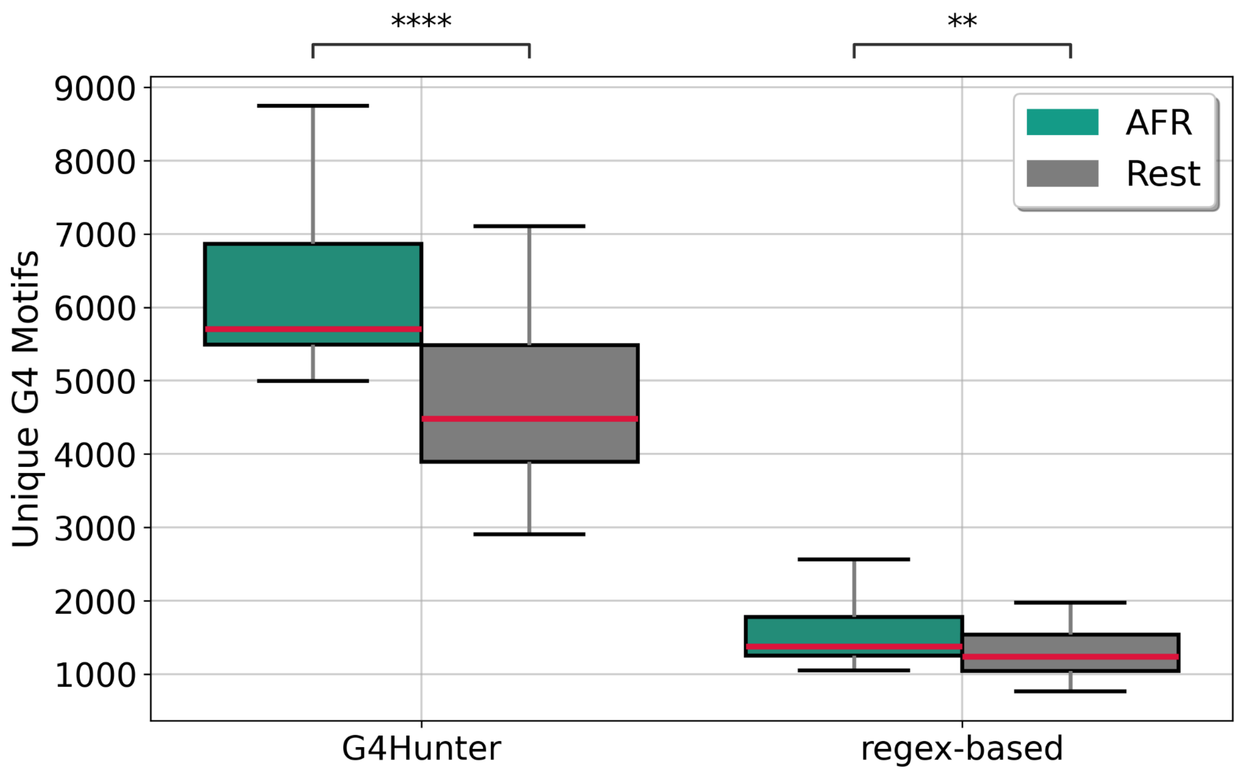

Supplementary Figure 3: Comparison of the number of unique G4 motif sequences identified in AFR haplotypes versus all other haplotypes, using both G4Hunter and regex-based methods. Independent two-tailed t-tests were performed, with p-values adjusted for multiple comparisons using the Bonferroni correction.

bioRxiv preprint doi: https://doi.org/10.1101/2025.06.17.660256; this version posted June 25, 2025. The copyright holder for this preprint (which was not certified by peer review) is the author/funder, who has granted bioRxiv a license to display the preprint in perpetuity. It is made available under aCC-BY 4.0 International license.

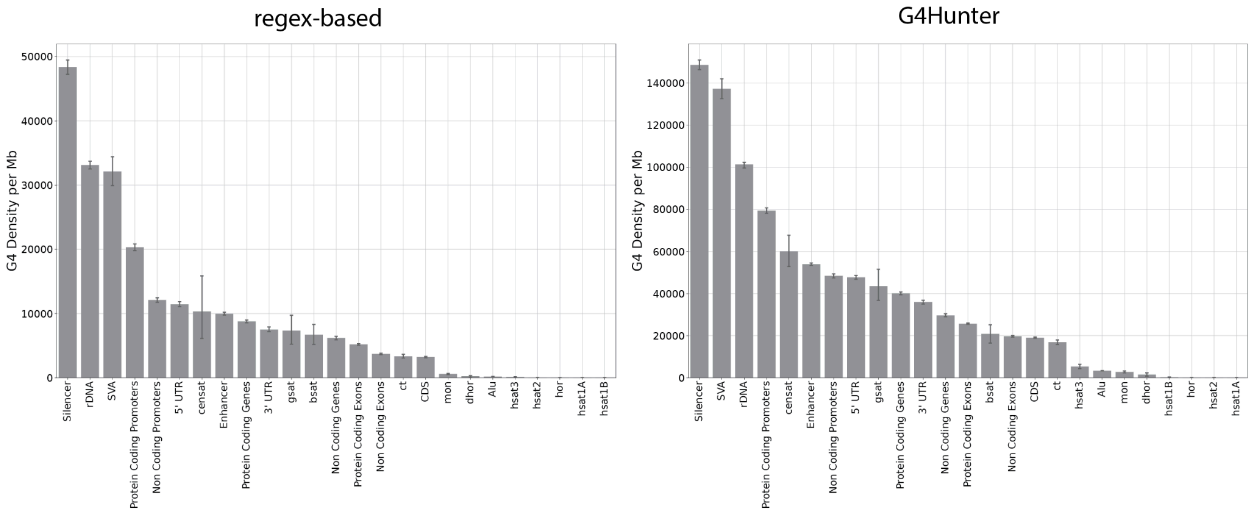

Supplementary Figure 4: G4 density per Mb across various genomic subcompartments for both G4Hunter and regex-based G4s. Telomeres were not included in the diagrams.

bioRxiv preprint doi: https://doi.org/10.1101/2025.06.17.660256; this version posted June 25, 2025. The copyright holder for this preprint (which was not certified by peer review) is the author/funder, who has granted bioRxiv a license to display the preprint in perpetuity. It is made available under aCC-BY 4.0 International license.

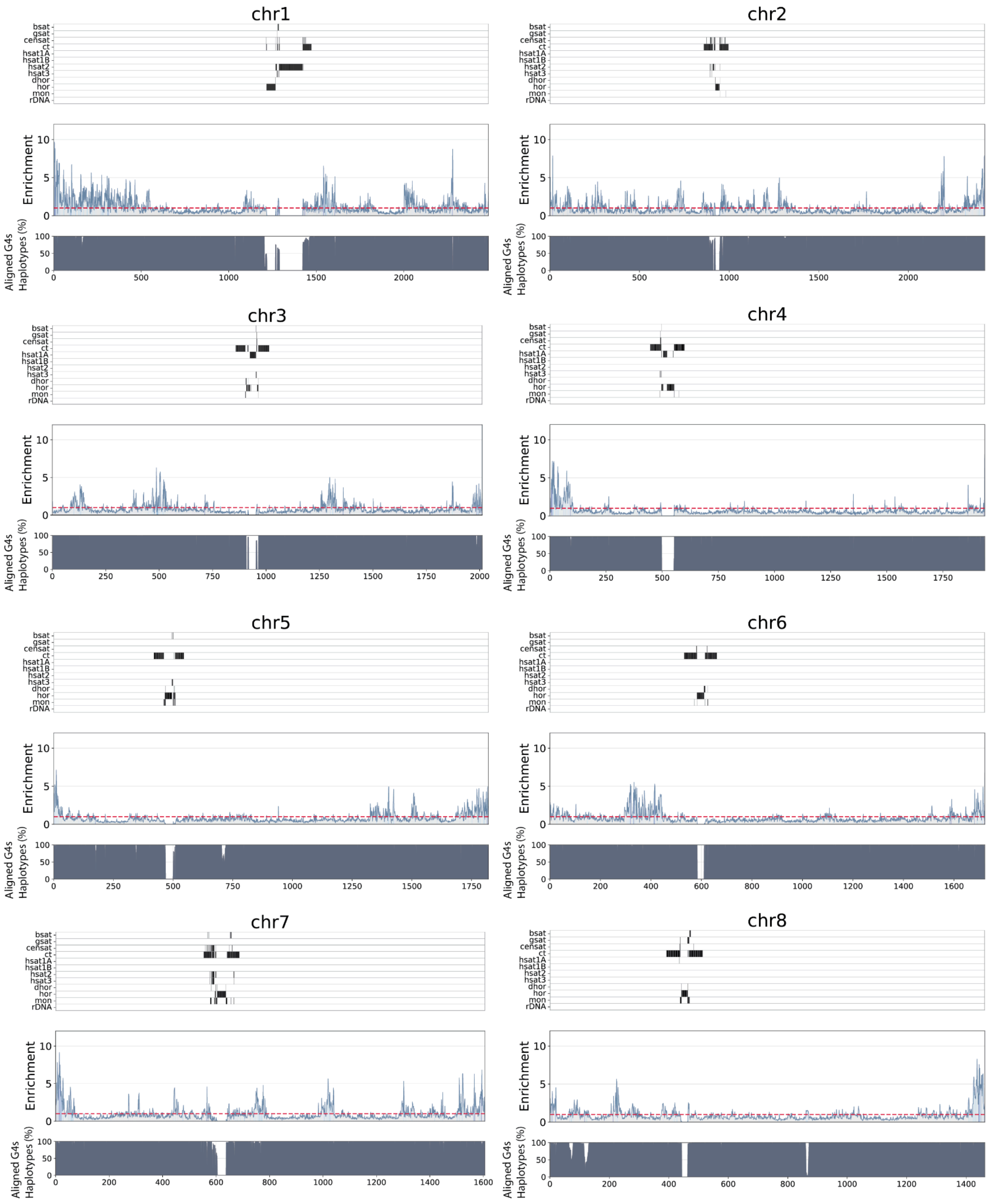

bioRxiv preprint doi: https://doi.org/10.1101/2025.06.17.660256; this version posted June 25, 2025. The copyright holder for this preprint (which was not certified by peer review) is the author/funder, who has granted bioRxiv a license to display the preprint in perpetuity. It is made available under aCC-BY 4.0 International license.

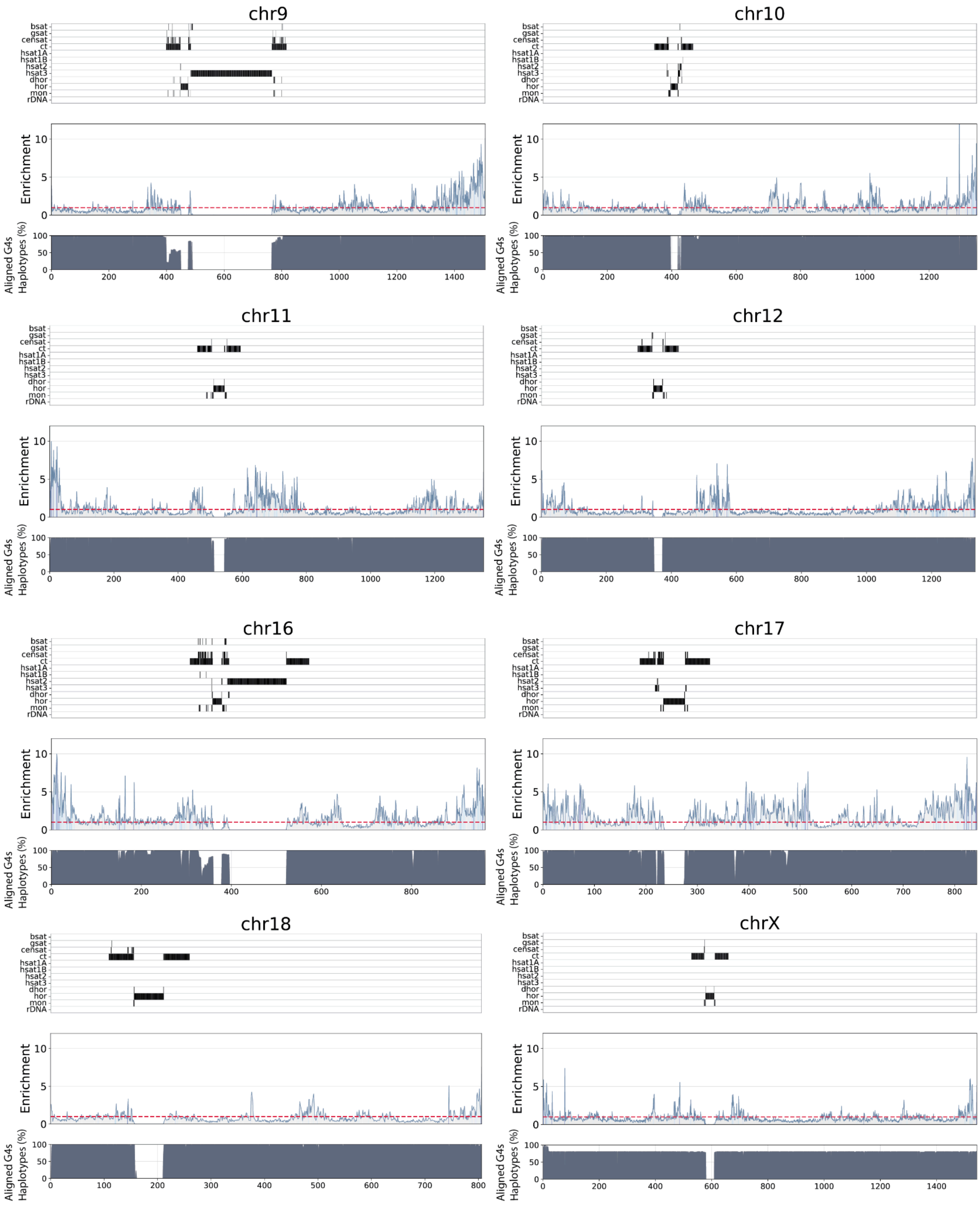

Supplementary Figure 5: Enrichment of G4s across human chromosomes, each one equipartitioned into 2,000 mutually exclusive regions. The area under the curve denotes the significance of the enrichment, with green representing a significant enrichment, and red a non-significant enrichment, when adjusting for GC-content. The top panel aligns the position of

bioRxiv preprint doi: https://doi.org/10.1101/2025.06.17.660256; this version posted June 25, 2025. The copyright holder for this preprint (which was not certified by peer review) is the author/funder, who has granted bioRxiv a license to display the preprint in perpetuity. It is made available under aCC-BY 4.0 International license.

centromeric and pericentromeric regions. The bottom panel illustrates, for each bin, the average percentage of haplotypes for which aligned G4s were conserved in a given bin. In a given bin, the white stripes represent either the absence of G4s or a low percentage of haplotypes. Repeats include inactive αSat HOR (hor), divergent αSat HOR (dhor), monomeric αSat (mon), classical human satellite 1A (hsat1A), classical human satellite 1B (hsat1B), classical human satellite 2 (hsat2), classical human satellite 3 (hsat3), β-satellite (bsat), γ-satellite (gsat), other centromeric satellites (CenSat), and centromeric transition regions (ct).

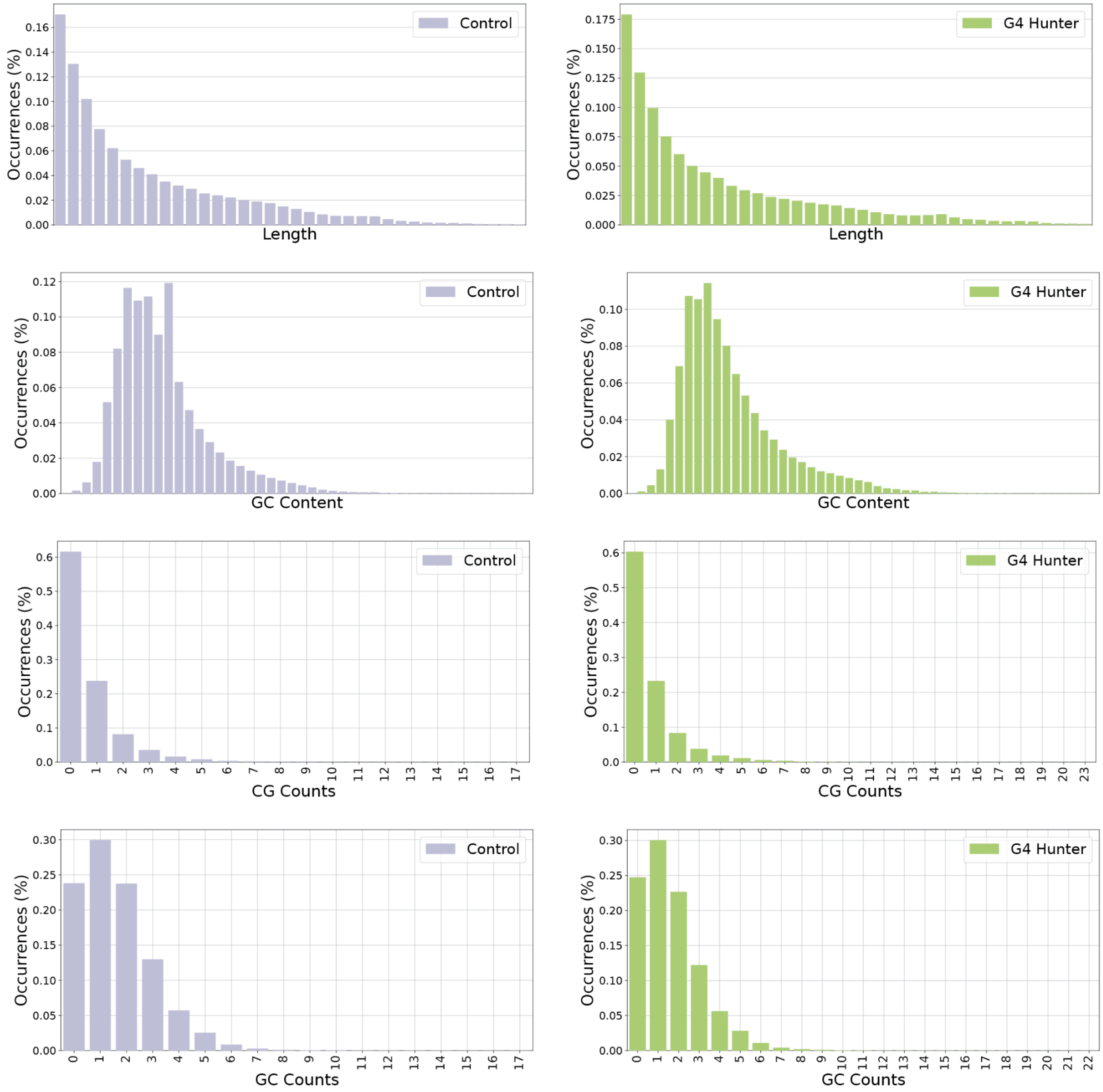

Supplementary Figure 6: **Nucleotide composition comparison between G4s from G4Hunter and G4 controls.**

bioRxiv preprint doi: https://doi.org/10.1101/2025.06.17.660256; this version posted June 25, 2025. The copyright holder for this preprint (which was not certified by peer review) is the author/funder, who has granted bioRxiv a license to display the preprint in perpetuity. It is made available under aCC-BY 4.0 International license.

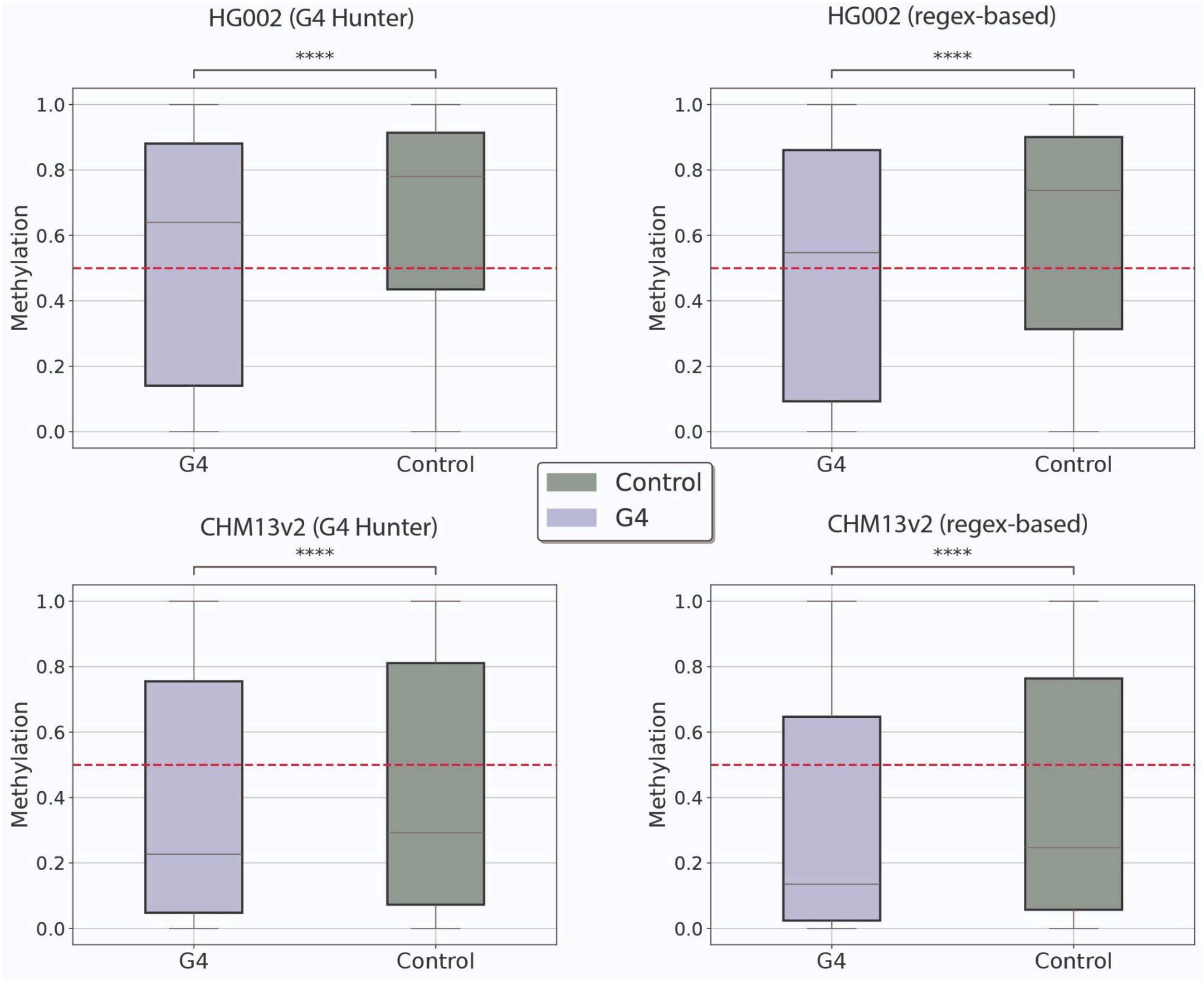

Supplementary Figure 7: **Comparisons of control group and G4 for both regex-based and G4Hunter extraction algorithms for genome-wide methylation profiles across lymphoblastoid and complete hydatidiform mold dell lines.** Results shown using Mann-Whitney U tests.

bioRxiv preprint doi: https://doi.org/10.1101/2025.06.17.660256; this version posted June 25, 2025. The copyright holder for this preprint (which was not certified by peer review) is the author/funder, who has granted bioRxiv a license to display the preprint in perpetuity. It is made available under aCC-BY 4.0 International license.

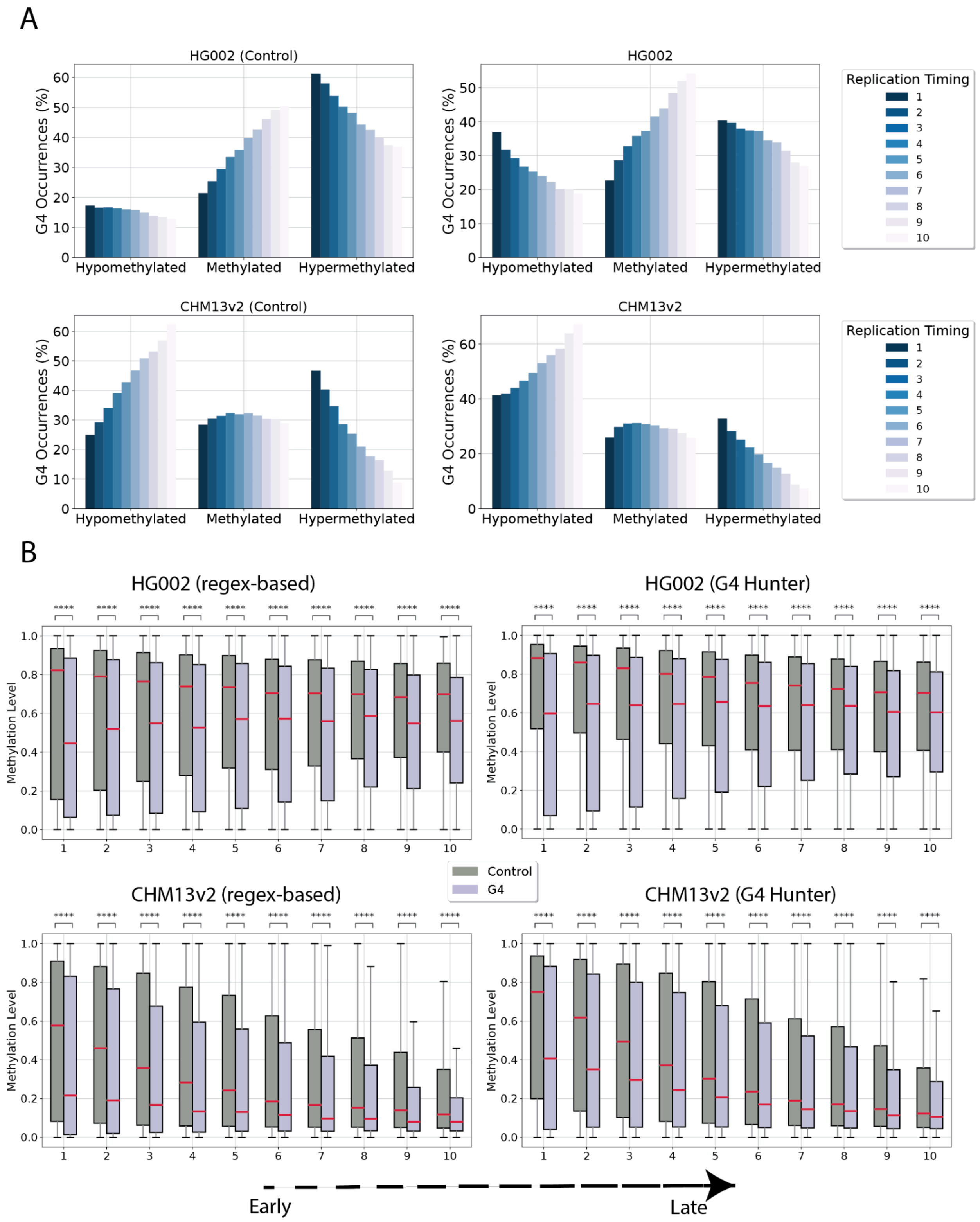

Supplementary Figure 8: **Replication timing and G4 methylation across two different cell lines. A**. Percentage of hypomethylated, methylated, hypermethylated controls and G4s across lymphoblastoid (HG002) and complete hydatidiform mold (CHM) cells for decreasing replication

bioRxiv preprint doi: https://doi.org/10.1101/2025.06.17.660256; this version posted June 25, 2025. The copyright holder for this preprint (which was not certified by peer review) is the author/funder, who has granted bioRxiv a license to display the preprint in perpetuity. It is made available under aCC-BY 4.0 International license.

timing. **B.** Comparisons of control group and G4 for both regex-based and G4Hunter extraction algorithms for decreasing replication timing for the lymphoblastoid and the CHM methylation patterns. Bonferonni adjusted comparisons.

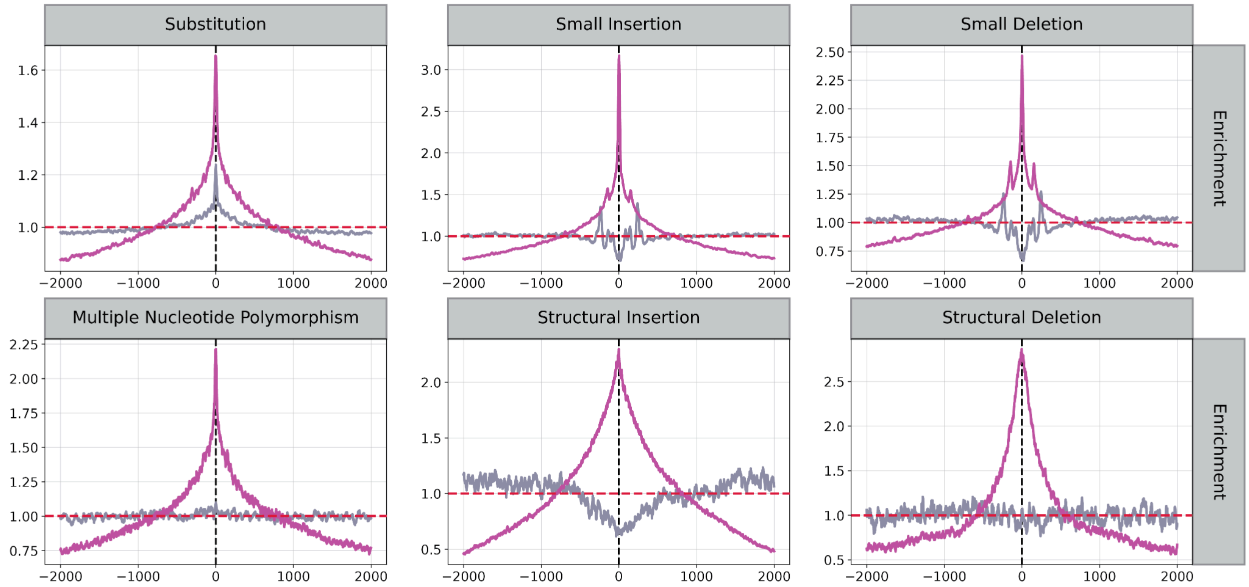

Supplementary Figure 9: Relative positioning of regex-based G4s (in magenta) and control group (gray) across various mutation loci.

bioRxiv preprint doi: https://doi.org/10.1101/2025.06.17.660256; this version posted June 25, 2025. The copyright holder for this preprint (which was not certified by peer review) is the author/funder, who has granted bioRxiv a license to display the preprint in perpetuity. It is made available under aCC-BY 4.0 International license.

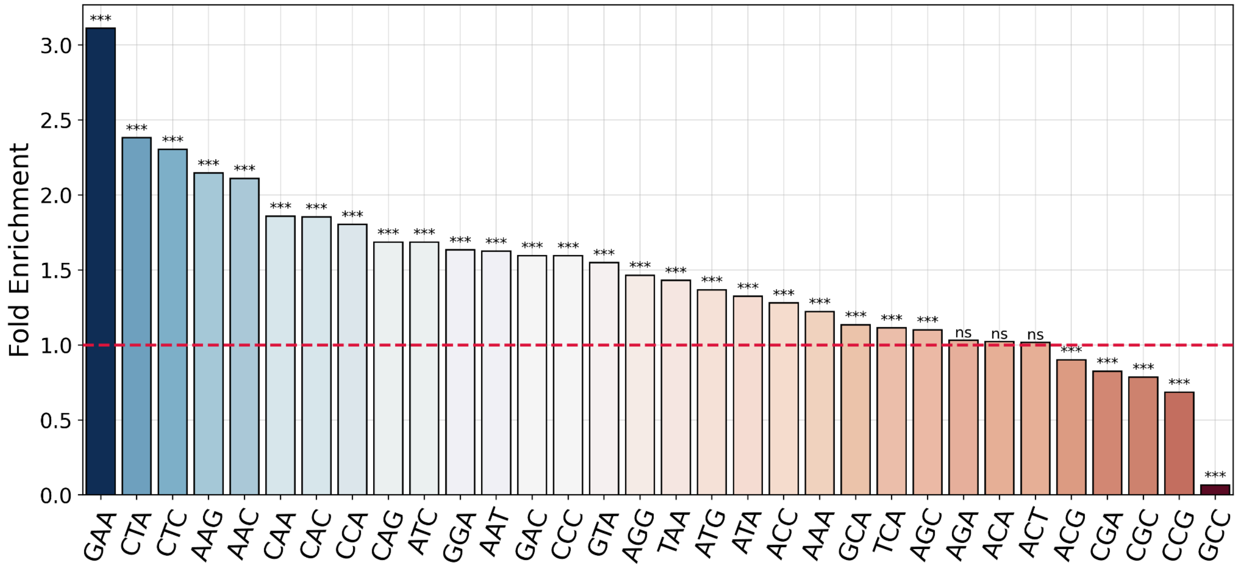

Supplementary Figure 10: Trinucleotide substitution model showing which trinucleotides are more frequently mutated at G4 motifs compared to matched control regions. Trinucleotides refer to a mutated base and its immediate 5' and 3' neighboring bases. This captures the influence of local sequence context on substitutions at G4s. Significance of the enrichment has been assessed using a two-tailed binomial test.

bioRxiv preprint doi: https://doi.org/10.1101/2025.06.17.660256; this version posted June 25, 2025. The copyright holder for this preprint (which was not certified by peer review) is the author/funder, who has granted bioRxiv a license to display the preprint in perpetuity. It is made available under aCC-BY 4.0 International license.

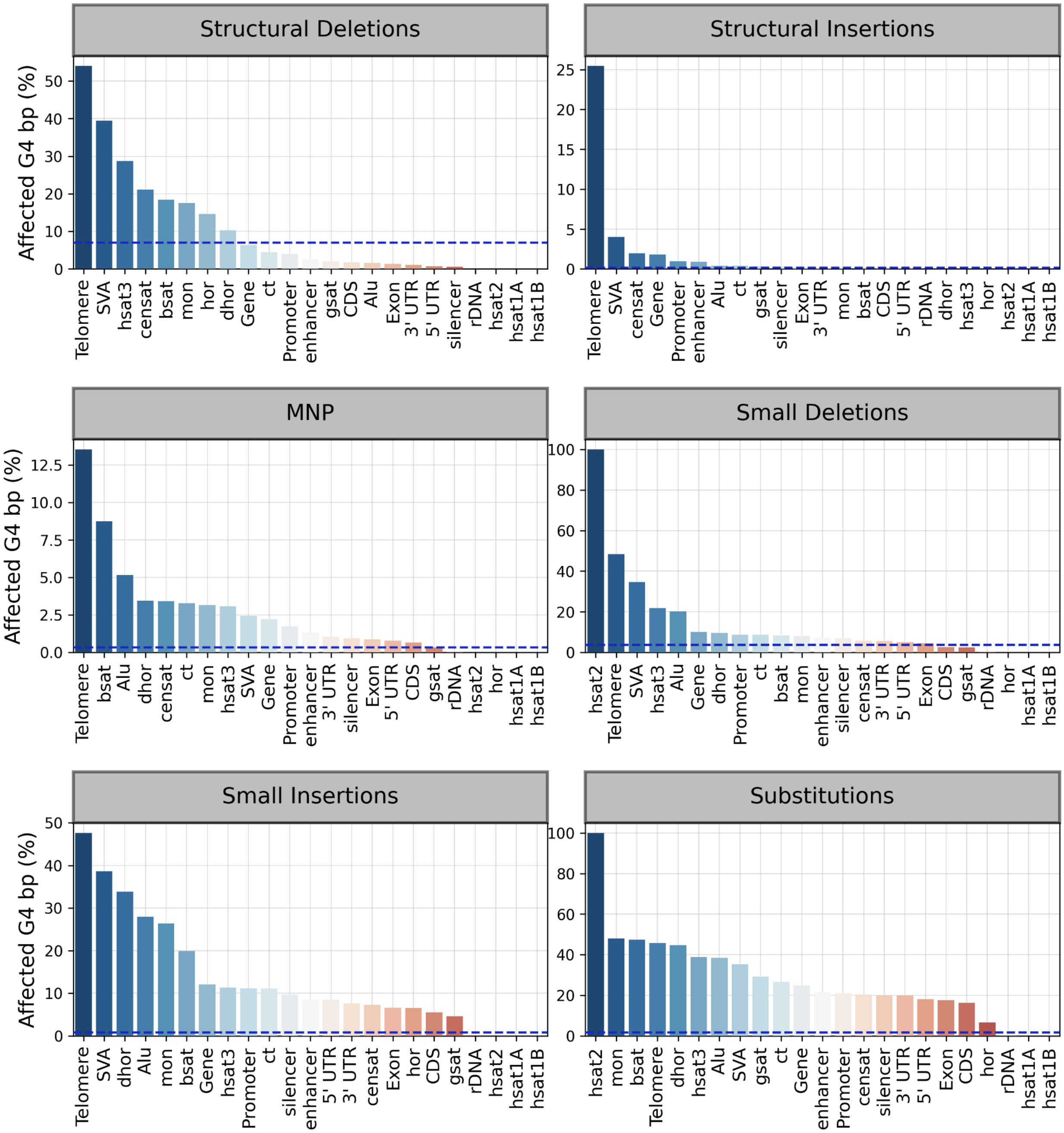

Supplementary Figure 11: Mutation rate of regex-based G4 base pairs across various genomic subcompartments of interest. In the Human Pangenome Reference Consortium, mutations falling in rDNA loci in many cases were masked.

bioRxiv preprint doi: https://doi.org/10.1101/2025.06.17.660256; this version posted June 25, 2025. The copyright holder for this preprint (which was not certified by peer review) is the author/funder, who has granted bioRxiv a license to display the preprint in perpetuity. It is made available under aCC-BY 4.0 International license.

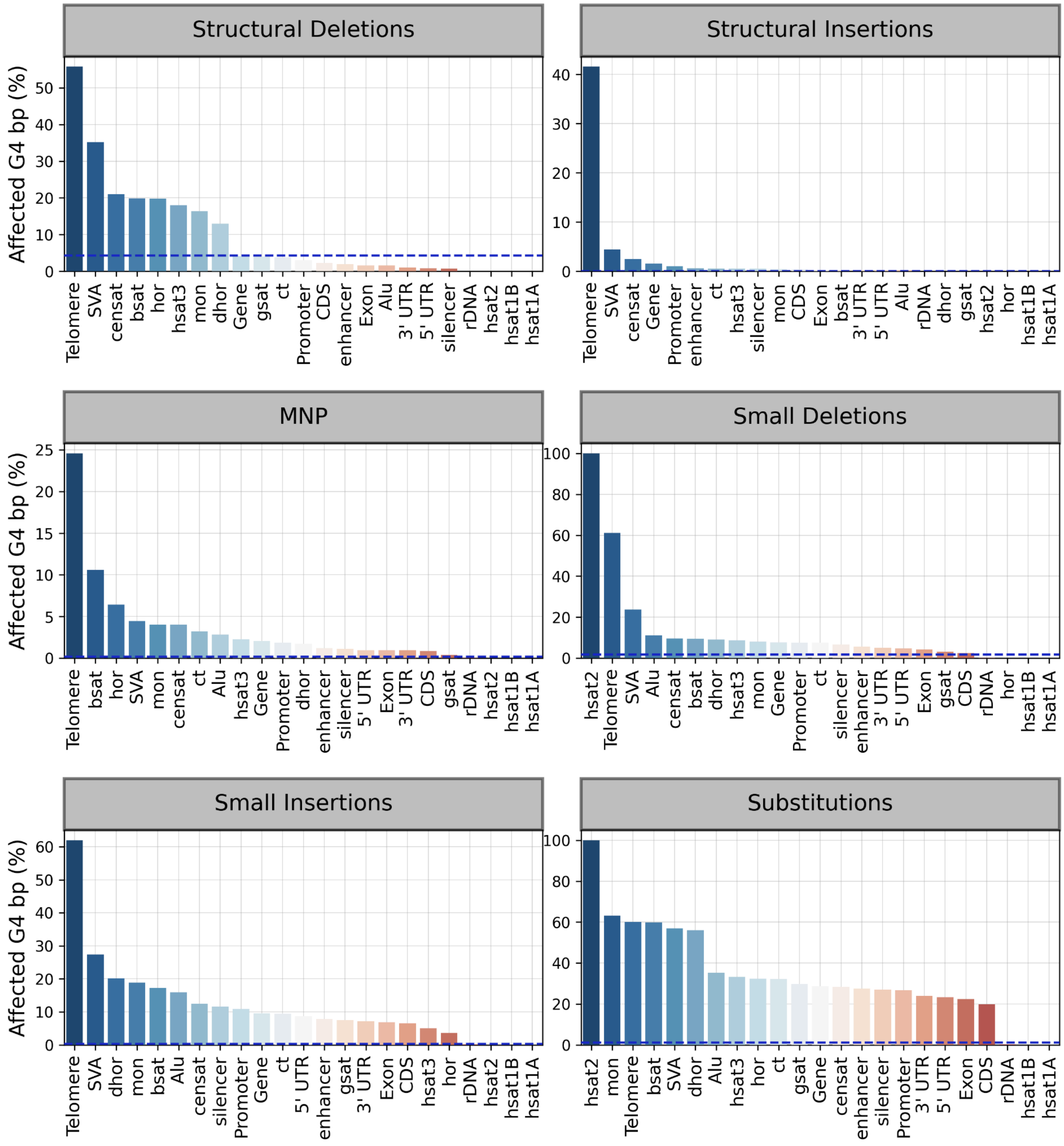

Supplementary Figure 12: Mutation rates of G4Hunter extracted G4 base pairs across various genomic subcompartments of interest. In the Human Pangenome Reference Consortium, mutations falling in rDNA loci in many cases were masked.

bioRxiv preprint doi: https://doi.org/10.1101/2025.06.17.660256; this version posted June 25, 2025. The copyright holder for this preprint (which was not certified by peer review) is the author/funder, who has granted bioRxiv a license to display the preprint in perpetuity. It is made available under aCC-BY 4.0 International license.

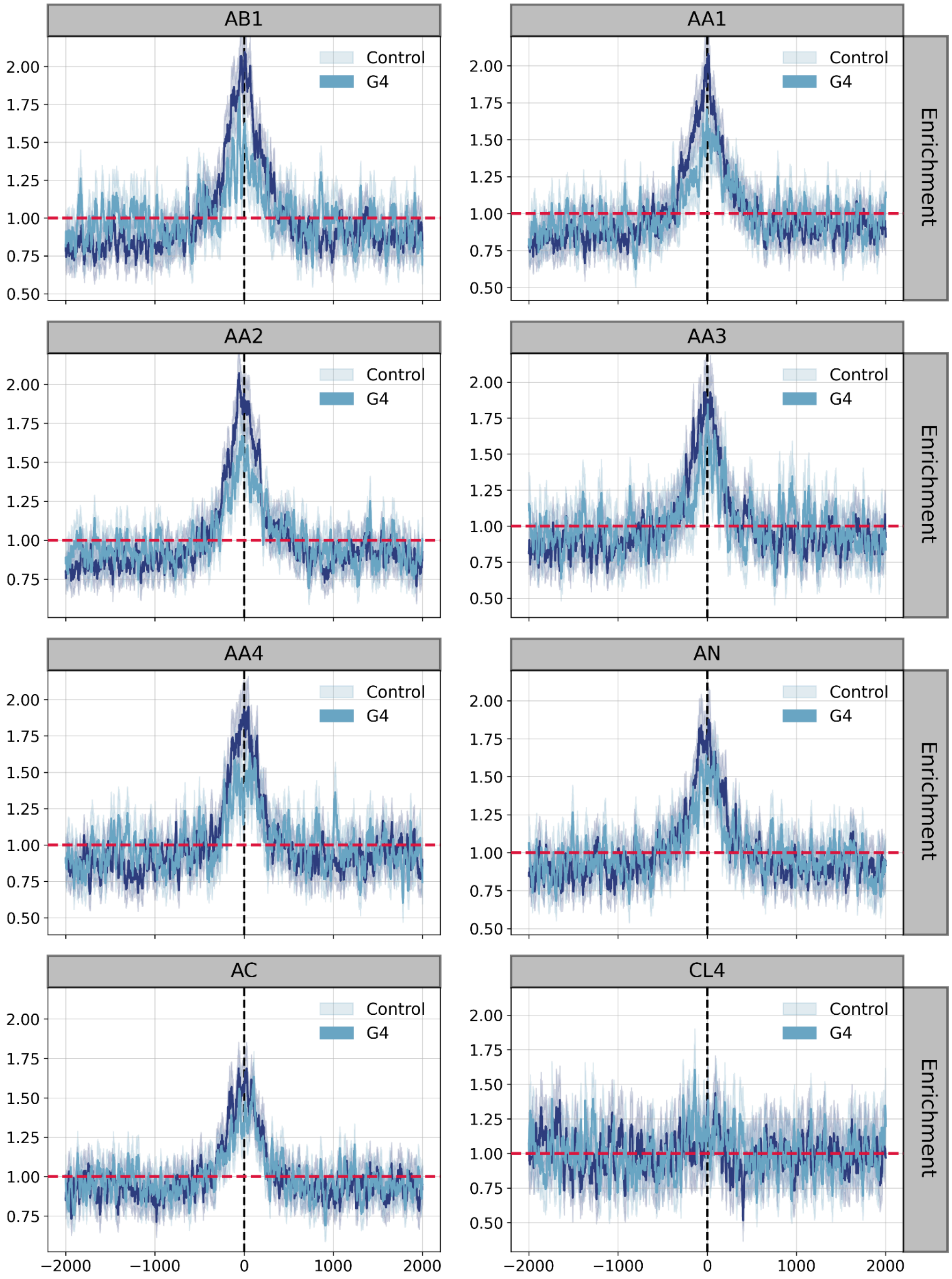

Supplementary Figure 13: PRDM9 regex-based algorithm 2kB density plot relative to PRDM9 loci for various alleles.

bioRxiv preprint doi: https://doi.org/10.1101/2025.06.17.660256; this version posted June 25, 2025. The copyright holder for this preprint (which was not certified by peer review) is the author/funder, who has granted bioRxiv a license to display the preprint in perpetuity. It is made available under aCC-BY 4.0 International license.

Supplementary Table 1: Most frequent canonical G4s within centromeric, pericentromeric and satellite regions. Selected sequences found in highly repetitive parts of the human genome based on G4 consensus and G4Hunter. Occurrences represent the genome-wide counts.

Name	Sequence (5'-3')	Compartment	Times appears in compartment	Genome-wide occurrences
G4Cen1	GGGAGGGAGGGAGGG	ct	925	21,834
G4Cen2	GGGTTAGGGTTAGGGTTAGGG	CenSat	1957	16,581
G4Cen3	GGGCTGAGGGTCAGGGAGAGGG	CenSat	148	153
G4Cen4	GGGTGGACGGGGGGGCCTGGTGGGG	rDNA	224	224
G4Cen5	CGGACGGGGCGGCTGGCCGGGCGGGGGCTGA	ct	185	1,845
G4Cen6	GTTGGAGGTGGGGCCTGGTGGGAGG	ct	62	981
G4Cen7	CGGCCTGGGAGGGTGGAGGGGAGTGTGGAACTG	bsat	53	53
